# HIV/AIDS in Conflict-Affected Countries, 2024–2025

**DOI:** 10.1007/s44197-026-00546-7

**Published:** 2026-05-08

**Authors:** Shahul H. Ebrahim, Udhayashankar Kanagasabai, Laura N. Broyles, Chantelle Owens, Stephanie Behel

**Affiliations:** Division of Global HIV and Tuberculosis Prevention, Global Health Center, Centers for Disease Control and Prevention, Atlanta, GA 30333 US

**Keywords:** AIDS control, Conflict, War, PEPFAR, Global Fund

## Abstract

**Background:**

Conflict reverses development gains and can intensify HIV challenges by driving displacement, poverty, sexual violence, and the collapse of health systems. These conditions may disrupt HIV testing, treatment initiation, and continuity of care. Given recent alerts of an increase in armed conflicts to historic highs, we conducted an ecological analysis of the strength of the relationships between conflict severity and HIV outcomes in select conflict-affected countries.

**Methods:**

We identified conflict-affected countries listed in all three data sources as of August 2025—the Armed Conflict Location and Event Data, Uppsala Conflict Data Program, and the World Bank. We extracted the most recent data from the Joint United Nations Programme on HIV/AIDS (UNAIDS) data on HIV incidence, prevalence, and the 95- 95-95 targets (95% of all people living with HIV have been diagnosed, 95% of those diagnosed are receiving antiretroviral therapy, 95% of those on antiretroviral therapy have achieved viral suppression). We used Pearson correlation to assess the strength of associations between conflict severity and HIV indicators.

**Findings:**

In the 22 countries included in the analysis, HIV prevalence ranged from 0.1% in Syria and Iraq to 11.6% in Mozambique. None of the countries met all three 95-95-95 targets based on available UNAIDS data, although Cameroon achieved 95% awareness of HIV status. We did not identify strong associations between conflict severity and HIV prevalence, incidence, or progress toward the UNAIDS 95-95-95 cascade targets.

**Interpretation:**

HIV indicators in conflict affected countries are within the range reported for other countries. We hypothesize that sustained external funding from the U.S. President’s Emergency Plan for AIDS Relief and the Global Fund to Fight AIDS, Tuberculosis, and Malaria, may have helped minimize potential effects of conflict on UNAIDS 95-95-95 cascade targets in countries with high preexisting HIV burden. Strengthening the monitoring of HIV indicators can help resource allocation and preparedness of health systems in the post-conflict reconstruction period.

**Supplementary Information:**

The online version contains supplementary material available at 10.1007/s44197-026-00546-7.

Conflict has often been described as development in reverse [1]. Underdevelopment fosters adverse health outcomes, a challenge particularly relevant for HIV both in terms of transmission and the continuum of care for people living with HIV. With respect to HIV transmission, the consequences of conflict (e.g., movement of armed groups and displaced persons, social disruptions, and widespread sexual violence) can create conditions conducive to the spread of HIV. Family separation, deepening poverty, and crippled healthcare systems further increase vulnerability to infection and limit health systems capacity for HIV diagnosis and treatment [2–5].

Coordinated global investments and innovation have led to major gains in control of the HIV epidemic. The U.S. President’s Emergency Plan for AIDS Relief (PEPFAR) and The Global Fund to Fight AIDS, Tuberculosis and Malaria (Global Fund) are the largest funding initiatives in the history of public health and have helped ensure scale-up of HIV prevention and treatment services in a range of low- and middle-income countries, including those with instability, displacement and underdevelopment [6].

There are many reasons to expect conflict to cause and predict high HIV prevalence and incidence, though measurement is a challenge during conflicts. HIV incidence reflects recent events; HIV prevalence is not time bound and can be impacted by various factors (e.g. HIV-related mortality which may cause HIV prevalence to decline). Among people living with HIV, conflict may impede HIV diagnosis, delay treatment initiation, and hinder treatment adherence and retention in care, due to factors including the inability to access available facility or community health services and medications, pharmacy stock-outs due to disrupted supply chains, or missed clinic visits due to destroyed infrastructure and/or unsafe travel circumstances. Stress and mental health challenges may also impede care-seeking behaviors and disrupt treatment adherence. However, evidence of these effects has not been consistent [7–9].

Given recent alerts of an increase in armed conflicts reaching a historic high in 2024 [10–12], we reviewed the status of the HIV epidemic in select conflict-affected countries, including any correlation between severity of conflict and HIV prevalence and incidence. Our analysis used most recent Joint United Nations Programme on HIV/AIDS (UNAIDS) data on HIV incidence and prevalence along with global and national 95-95-95 target progress that refers to (1) 95% of people with HIV infection know their HIV status, (2) 95% of those diagnosed with HIV are on antiretroviral treatment, and (3) 95% of those on antiretroviral treatment have achieved viral suppression, respectively [13].

## Methods

We obtained the list of conflict-affected countries from three sources as of August 2025: the Armed Conflict Location and Event Data (ACLED) [14], the Uppsala Conflict Data Program (UCDP), [15] and the World Bank [16]. ACLED is a global data collection and crisis mapping system that documents the dates, actors, locations, fatalities, and types of all reported political violence and protest events.

Of these sources, ACLED provides a conflict severity index ranking and categorizes conflict into three levels: turbulent (the lowest level indicated by conflict severity index 31 to 50), high (the intermediate level indicated by conflict severity index 11 to 30) or extreme (the highest level indicated by conflict severity index of 1 to 10) (Panel 1). UCDP, based at the Department of Peace and Conflict Research at Uppsala University in Sweden, is the world’s leading provider of organized violence data and the longest-running data project on civil war. The World Bank’s Fragility, Conflict, and Violence datasets support analysis of displacement, food insecurity, and other aspects of fragility and conflict. Due to differing definitions of conflict (Panel 1) and variations in country designations (e.g., Palestine is listed as a “non-member observer state” by the UN), the number of countries listed varies across sources (ACLED: 50; UCDP: 38; World Bank: 39).

**Table:** 

Panel 1. Definition of conflict in various data sources
ACLED [14]: conflict is a politically violent event, a single altercation where force is used by one or more groups toward a political end. The ACLED Conflict Severity Index is calculated using four indicators over the past 12 months – deadliness, danger to civilians, geographic diffusion, and the number of armed groups. Scores from each indicator are combined into an overall index rank called the conflict severity index and categorized as *Extreme (rank 1 to 10)*, *High (rank 11 to 30)*, or *Turbulent* (31 to 50) levels of conflict. ACLED=Armed Conflict Location and Event Data.
UCDP [15]: an armed conflict is a contested incompatibility that concerns the government and/or territory where the use of armed force between two parties, of which at least one is the government of a state, results in at least 25 battle-related deaths in one calendar year. UCDP= Uppsala Conflict Data Program.
World Bank [16]: conflict is a situation of acute insecurity driven using deadly force by a group, including state forces, organized non-state groups, or other irregular entities, with a political purpose or motivation.

For this analysis, we selected countries listed as ‘in conflict’ in all three data sources (supplementary Figure S1, Countries included in the three data sources). For those, we analyzed the most recent five HIV epidemiological indicators from UNAIDS (Table 1) [13], including incidence, prevalence, and the 95-95-95 targets. Both UCDP and World Bank data subsumes Palestine under Isreal, and for consistency we included only Isreal. These indicators are modeled by UNAIDS for 2024 from self-reports of national data from country data reporting systems and population-based surveys or surveillance systems, using the most recent available input data with a 2024 cutoff. To contextualize the HIV burden within broader development trends, we also obtained select development indicators from the World Bank (Table 1).

**Table 1:** HIV/AIDS and select human development indicators in conflict-affected countries, 2024*

Conflict characteristics			UNAIDS HIV indicators [13]					Development indicators [17]		
Country/ Territory	Conflict Index Rank (ACLED) [14]	Conflict Index Level (ACELD) [14]	Incidence Per 1000 population	Prevalence (%)	% of PLHIV aware of their infection	% of PLHIV with diagnosed infection on ART	% of PLHIV on ART recipients with suppressed viral load	Gross Domestic Product (USD)	Health Expenditure Per Capita (USD)	Human Development Index
Myanmar [1, 2]	1	Extreme	0.21	0.9	77	77	74	1233.1	57.9	0.609
Syria [1]	2	Extreme	0.01	0.1	77	57	ND	1051.6	34.3	0.564
Ukraine [1, 2]	4	Extreme	ND	ND	75	83	94	4199.6	369.9	0.779
Nigeria [1, 2]	5	Extreme	0.26	1.2	85	85	82	1596.6	90.9	0.56
Yemen [1]	7	Extreme	0.03	0.1	38	28	ND	477.4	38.1	0.47
Iraq	8	Extreme	0.01	0.1	68	46	ND	5565.1	254.6	0.695
DRC [1, 2]	9	Extreme	0.13	0.6	87	86	77	627.5	24.3	0.522
Haiti [1, 2]	12	High	0.58	1.7	87	84	71	414.7	80.65	0.554
Afghanistan [1]	13	High	0.03	0.1	27	9	6	415.71	80.65	0.496
Burkina Faso [1, 2]	13	High	0.08	0.6	83	77	71	882.6	56.75	0.459
Mali [1, 2]	15	High	0.28	0.9	62	58	ND	869.2	29.4	0.419
Sudan [1]	16	High	0.09	0.1	40	23	ND	2183.4	31.5	0.511
Cameroon [1, 2]	24	High	0.36	2.6	95.8	92.3	89.2	1736.8	71.9	0.588
Ethiopia [1, 2]	25	High	0.08	0.8	90	85	75	1272	27	0.497
Niger [1, 2]	30	Turbulent	0.06	0.2	78	70	51	642.9	26.5	0.419
South Sudan [1, 2]	31	Turbulent	0.79	1.9	51	47	ND	ND	49.4	0.388
CAR [1]	34	Turbulent	1.5	3.4	ND	ND	ND	495.9	47.9	0.414
Burundi [1, 2]	35	Turbulent	0.1	0.9	92	91	81	191	24	0.439
Mozambique [1, 2]	37	Turbulent	3.2	11.6	89	91	80	622.9	49.4	0.493
Israel * [1]	42	Turbulent	ND	ND	ND	ND	ND	52642.4	4223.9	0.919
Chad [1]	47	Turbulent	0.2	1	71	61	ND	680.6	40.1	0.416
Libya	39	Turbulent	0	0.2	90	54	ND	6172.8	278.3	0.721

*ACLED=Active Conflict Location Event Data; rankings are updated frrequenly, we releid on data at the time of analysis. ART= antiretroviral treatment. ^1^GFTAM fund recipient. ^2^PEFPAR fund recipient. ND = no data available on UNAIDS. ART=antiretroviral treatment. *ACLED includes Isreal and Palestine (which recieves GFTAM funds), however Palestine is subsumed under Israel in UCPD and World Bank data. Therefore, to be consistent with all three data sources, we report Isreal. PLHIV=People living with HIV. Data Sources, ACLED [14], UNAIDS [13], World Bank [17]. Please note that these data sets are updated an ongoing basis, and current listing and rankings may differ from August 2025, when this work was completed.

We used Pearson correlation to assess the strength and direction of linear associations between ACLED conflict severity rankings and each of the five HIV indicators. We considered a Pearson correlation coefficient ( r) = > 0.50 as a strong association. We did not conduct a stratified analysis by HIV/AIDS funding support as 20 of 22 countries received either PEPFAR or Global Fund support.

## Findings

Our analysis included 22 conflict-affected countries. Of these 22 countries, ACLED categorizes conflict index level in seven as extreme, eight as high, and seven as turbulent (Table 1). Of these 22 countries, 20 received PEPFAR and/or Global Fund funding support (13: PEPFAR and Global Fund; 7 Global Fund only) (Table 1).

Overall, HIV prevalence and incidence data were available for 19 of the 22 countries (Table 1); in these, HIV prevalence varied from 0.1% (Syria, Iraq) to 11.6% (Mozambique) (Table 1).

**Fig. 1:**
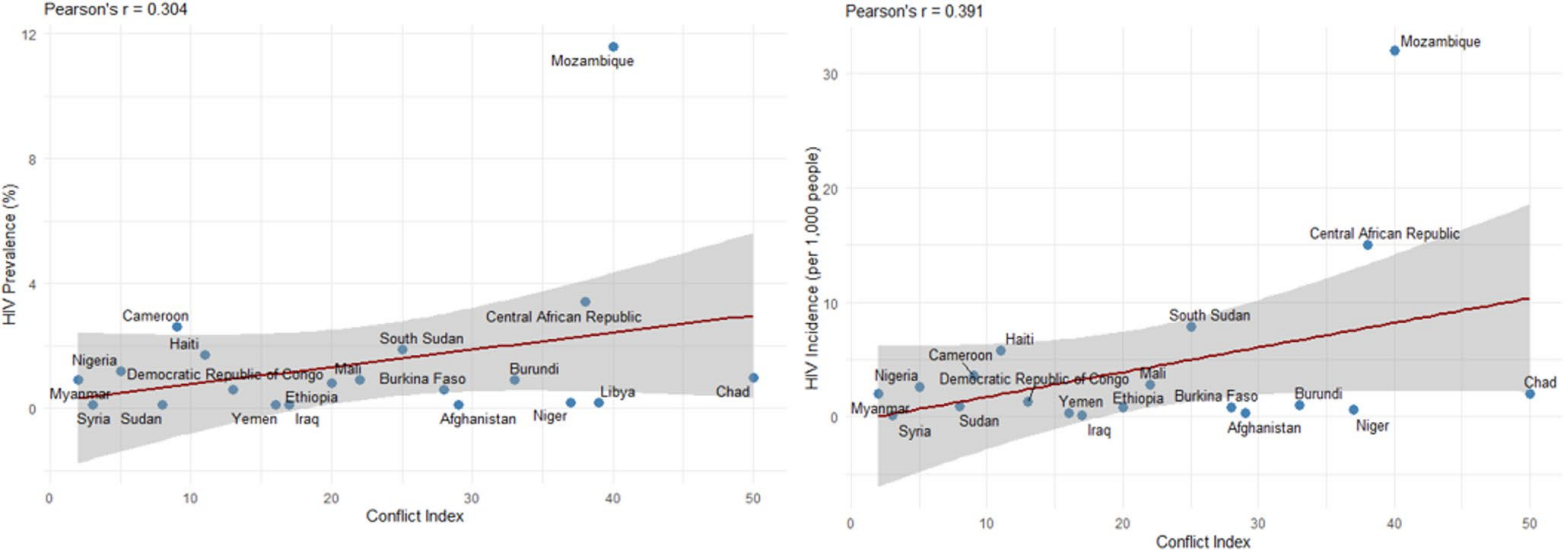
Correlation of conflict index rank with UNAIDS data [13] on prevalence and incidence of HIV in select conflict-affected countries, 2024–2025*. *Data sources: ACLED [14], UNAIDS [13]. Incidence, *r* = 0.304, *p* = 0.192. Prevalence, *r* = 0.391, *p* = 0.11

Overall, countries in the worst ACLED conflict category (extreme = conflict severity index 1 to 10) had the lowest HIV prevalence (range 0.1 to 0.9%), while countries in the lowest category (turbulent= conflict severity index 31 to 50) had the highest HIV prevalence (range 1.0% to 11.6%) (Table 1). In the 19 conflict-affected countries with incidence data, HIV incidence (per 1000 population) ranged from 0.01 (Syria, Iraq) to 0.79 (South Sudan) (Table 1). We did not detect a strong relationship between HIV incidence or prevalence and conflict severity index (incidence, *r* = 0.304, *p* = 0.192; prevalence, *r* = 0.391, *p*=0.0.11. Figure 1).

Of the 22 conflict-affected countries, 20 countries had available data for the first two HIV epidemic control indicators (percentage of PLHIV aware of their infection status and, percentage of diagnosed people living with HIV (PLHIV) on antiretroviral treatment) (Table 1). For the third indicator (percentage of antiretroviral recipients who were virally suppressed), data were available for 12 countries.

No conflict-affected country has achieved the 95% goal in any of the three epidemic control indicators, except for Cameroon, where 96% of PLHIV are aware of their infection status (Table 1). The percentage of people who knew their HIV status is the lowest in Afghanistan (27%), Yemen (38%), and Sudan (40%) – three countries with extreme or high levels of conflict and no PEPFAR support (Table 1).

**Fig. 2:**
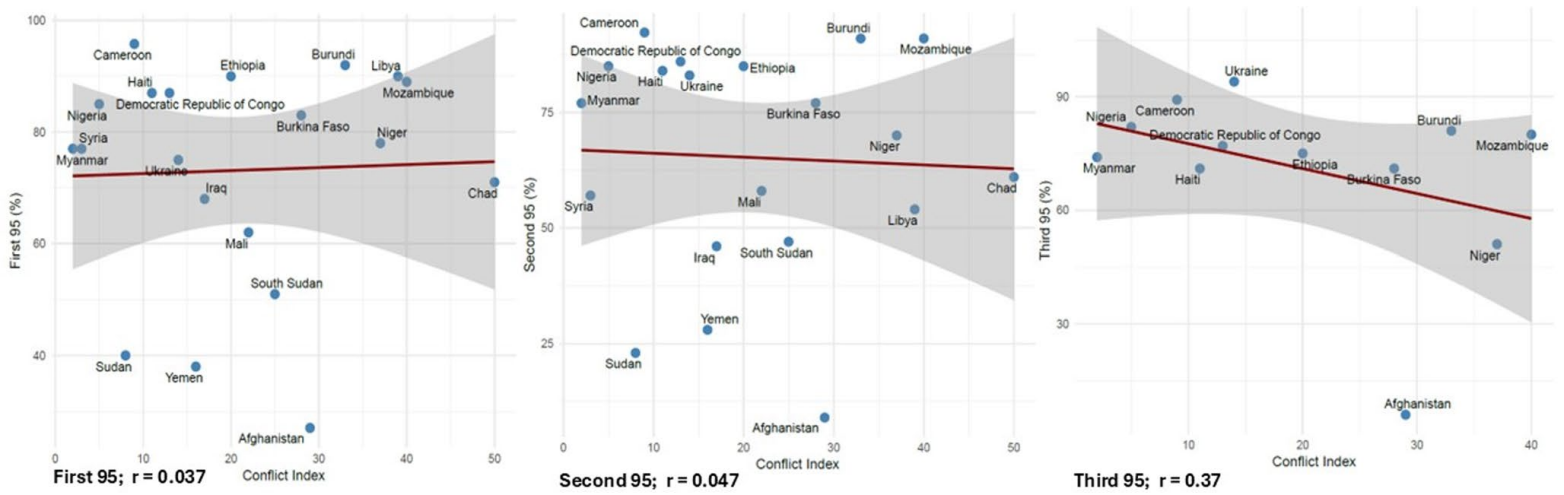
Correlation between conflict index rank and UNAIDS [13] treatment cascade targets in select conflict-affected countries, 2024–2025*. *UNAIDS Targets [13]. First 95 = 95% of all people living with HIV have been diagnosed (*r* = 0.037, *p* = 0.877). Second 95 = 95% of those diagnosed are receiving antiretroviral therapy (*r* = 0.47, *p* = 0.845). Third 95 = 95% of those on antiretroviral therapy have achieved viral suppression (*r* = 0.37, *p* = 0.231). Data sources: ACLED [14], UNAIDS [13]

Irrespective of the conflict severity level, countries with PEPFAR support showed a higher percentage of people living with HIV who knew their HIV status (1st 95) (Table 1). Of the five countries that had only Global Fund support that had data on the first 95, three countries had less than 50% who knew their status (Table 1). Similar trends were observed for the second and third indicators (Table 1). We did not detect any statistically significant relationship between the three UNAIDS HIV/AIDS targets and conflict severity (first 95, *r* = 0.037, *p* = 0,877; second 95, *r* = 0.047, *p* = 0.845; third 95, *r* = 0.37, *p* = 0.231. Figure 2).

Among the conflict-affected countries in this analysis, Ukraine and Israel had the highest levels of development as determined by gross domestic product (GDP), per capita health expenditure, and human development index. Overall, 40% of the conflict-affected countries had a human development index below 0.5. Per capita annual health expenditure was below USD 100 in all countries except Ukraine and Israel.

## Discussion

Among the 22 conflict-affected countries, we detected no clear relationship between conflict and HIV/AIDS epidemic control targets or epidemiologic indicators. Generally, the HIV burden was slightly lower in countries categorized as extreme on the conflict severity index than in those categorized as turbulent on the conflict severity index. Though not statistically significant, knowledge of HIV status and treatment coverage in diagnosed PLHIV was the lowest in countries with a high conflict severity index. HIV indicators in conflict-affected countries fell within the range reported [13] for other countries.

Earlier reports have also demonstrated a lack of consistent pattern in the effect of conflict on HIV. [7–9, 18–20] Conflict may impact HIV/AIDS indicators differently. HIV testing can be disrupted during times of conflict, in ways likely impacted by severity of the conflict, which will then impact prevalence and incidence being reported. HIV transmission appears to occur more during periods of peace compared to during or immediately following conflict,^18^ whereas treatment and retention are more likely to be affected during conflict. These factors are more significant if such countries have other preexisting health and development challenges [8, 20]. For example, Mozambique experienced a long civil war from 1977 to 1992, followed by peace, and the current conflict in Northern Mozambique began in 2017. HIV prevalence increased during the period of peace (1992–2017) following the conflict, as has been reported earlier in a larger selection of conflict-affected countries [19].

Of note, the decades around the 1990s were a period of rapid increase in HIV infections in east and southern Africa due to the lack of interventions, and limited data availability, confounding the associations between peace and conflict. Furthermore, HIV prevalence may be impacted by movement of internally displaced people and refugees between geographic regions with different HIV incidences, so that values measured in each location may partly reflect transmission which took place elsewhere. It is also possible that HIV transmission occurred in a time frame outside of the conflict time frame. In resource poor countries, compounded by conflict, precise measures of recency of infection are likely to be unavailable, and sometimes HIV prevalence data obtained post conflict may be a mix of infections occurring at various time periods. Over 4 million people were displaced from Mozambique during the civil war [21]. Civilians fled mostly to Malawi and Zimbabwe, other rural areas, and cities, followed by repatriation between 1992 and 1994.

The “paradoxical protective effect” of conflict on HIV indicators is perhaps most evident in the Democratic Republic of the Congo (DRC). In the DRC, though genomic evidence indicated the emergence of HIV much earlier than documented in the rest of Africa,^21^ the three decades of continuous conflict may have minimized extensive transmission of HIV beyond the southern province of Haut Katanga. Persistent conflict and insecurity in eastern DRC disrupted transport and trade infrastructure, limiting movement of people and further spread of HIV from southern DRC to most other provinces [22, 23].

Though conflict-related sexual violence and commercial sex work may precipitate HIV transmission, data from several countries have indicated the opposite. Data from seven countries prior to 2007 did not show an increase in prevalence of HIV infection during periods of conflict, irrespective of the HIV prevalence when conflict began [7, 24]. Work in Burundi found that the relationship between conflict and HIV was a function of pre-existing power dynamics and social norms between men and women that also regulate sexual life and determine critical female vulnerabilities [24]. HIV prevalence in urban areas affected by conflict decreased in Burundi, Rwanda, and Uganda at similar rates to urban areas unaffected by conflict in their respective countries [7, 24, 25] Prevalence in conflict-affected rural areas remained low and fairly stable in these countries [7]. Of the 12 sets of refugee camps, nine had a lower prevalence of HIV infection and two a similar or higher prevalence than their respective host communities [7]. Access to HIV testing and health care may be better in some refugee camps compared to host communities, while in other settings the opposite conditions may apply.

Reports that included adverse outcomes of conflict on HIV include diagnostic delays of HIV in Ukraine, antiretroviral stockouts and loss to follow up in Yemen, both reduction in public sector health system and increase in involvement of nongovernmental organizations in Cote d’Ivoire, and change from local to geographically generalized transmission of HIV in Libya and Somalia without substantial increase in overall national burden, [26–29]. Conversely, a systematic review found lower rates of non-adherence, loss to follow-up, virologic non-suppression, and mortality in conflict zones compared to those reported from politically stable and well-resourced regions in Sub-Saharan Africa during 2002 to 2022 [8].

Given the multidimensional transmission dynamics of HIV (e.g., mother-to-child (vertical), injection drug use, sexual behavior, blood transfusion and other nosocomial routes) conflict may not uniformly influence the various HIV transmission routes, thus limiting conflicts’ impact on HIV. Health systems’ ability to respond and remain resilient during conflicts also varies, as do displacement of people and impact on health systems infrastructure [30]. Other contributing factors that impact HIV response include socioeconomic conditions, pre-conflict HIV burden, the availability of external funding for HIV control, and the presence of non-state actors [5, 7, 8, 30]. Tools such as multi-month prescriptions improve continuity of treatment during conflicts. Countries with lower levels of peace and higher corruption had lower ART coverage (*P* > 0.001).^19^ Countries with a global peace index > 2.5 all had ART coverage of > 40% [20]

Taken together, we posit at least three interconnected factors at play in discerning the relationship between conflict and HIV. First is the variation in baseline HIV burden across geographic regions that may affect HIV natural history during conflicts. The countries of the middle eastern region, a region with several extreme conflicts, had historically the lowest burden of HIV [31]. Second is the natural history of HIV; the incident HIV infections peaked in 1997 and deaths in 2004 and a downward trend is observed [31]. Due to measurement and reporting challenges including reduced access to testing and surveillance, denominator instability due to displacement, reported data and assumptions used in UNAIDS spectrum estimates are unlikely to be reflect the true burden. Further, micro-trends in HIV burden in select geographic areas, especially those occurring during conflicts, may not always be sufficient to alter national estimates (for example, Libya [29]) to a level detectable in summary data analysis. Finally, external HIV/AIDS funding support in conflict-affected countries may have minimized conflict’s effects on HIV. In the absence of such support, resource-poor countries, especially those with relatively low HIV burden, likely would have low achievement on the three 95s [13] regardless of conflict, as low prevalence makes HIV control a lower public health priority [32]. Many of these countries would not have baseline HIV prevalence data but for the efforts of PEFAR and the Global Fund support.

Findings from this report should be interpreted within the scope and limitations of the analytical methods, as the availability of HIV indicator data was not uniform for the 22 countries included, and variations existed in periods of data collection. The datasets are dynamic and updated frequently, and we restricted the analysis to data available in 2025. The variation in definition of conflict among the three data sources on conflict is an additional limitation, as only 22 countries are included in all three data sources. The inclusion/exclusion criteria used by the three conflict data sources may be controversial. We refrained from arbitrary inclusion or exclusion of countries to minimize further bias. As conflicts may be geographically localized, country level estimates of HIV data may not reflect transmission dynamics in specific geographic areas affected by conflict. Also, some countries have been in conflict during a period of substantial advances in access to evidence-based HIV interventions, impact of which may not be fully captured by national level aggregate data. For the countries with available HIV data, data collection and reporting may be impaired during conflicts, affecting completeness and accuracy. In addition, development indicators as well as behavioral and social dynamics underpinning HIV transmission vary across countries, limiting comparisons of the impact of conflicts across countries. Furthermore, heterogeneity in data collection timing and reporting, and any inaccuracies introduced in the modeling process when projecting forward may affect comparability and recency.

In summary, our ecological analysis found no strong association between conflict and HIV/AIDS. The increasing trend in conflict highlights the need for monitoring and reporting of HIV indicators pre- and post-conflict. Such data can inform assessments of the conflict-health link and help resource allocation and preparedness of health systems.

## Supplementary Information

Below is the link to the electronic supplementary material.

Supplementary File 1 (DOCX 161 KB)

## Data Availability

All data used in this article are public use data. Ethics, Consent to Participate, and Consent to Publish declarations: not applicable.
