# Supplementary material for: HIV/AIDS in Conflict-Affected Countries, 2024–2025
Source: J Epidemiol Glob Health. 2026 May 8;16(1):79. doi: 10.1007/s44197-026-00546-7 (PMC13333569; doi:10.1007/s44197-026-00546-7)
Supplement: Supplementary file 1 [file 44197_2026_546_MOESM1_ESM.docx]

### HIV/AIDS in Conflict-Affected Countries, 2024-2025

### *Shahul H. Ebrahim, [sbe2@cdc.gov](mailto:sbe2@cdc.gov), ORCID#0000-0002-0746-2326

### Udhayashankar Kanagasabai, [nqy2@cdc.gov](mailto:nqy2@cdc.gov), 

### Laura N Broyles, zjt5@cdc.gov

### Chantelle Owens, cfo7@cdc.gov

### Stephanie Behel, zmd7@cdc.gov

### Division of Global HIV and Tuberculosis Prevention

### Global Health Center

### US Centers for Disease Control and Prevention, Atlanta, GA 30333, USA 

### Word count, narrative 2,582

### *Corresponding

Diagram, venn diagram  AI-generated content may be incorrect.

Supplementary Figure 1. Conflict affected countries listed by three independent data sources, 2024. World Bank ([Fragility, Conflict and Violence | World Bank Group](https://www.worldbank.org/ext/en/topic/fragility-conflict-and-violence),) ACLED (Armed Conflict Location & Event Data, [ACLED](https://acleddata.com/)) and UCPD (Uppsala Conflict Data Program [UCDP – Department of Peace and Conflict Research – Uppsala University](https://www.uu.se/en/department/peace-and-conflict-research/research/ucdp)). The full circle in the center lists countries listed in all three data sources.
